# Objective Assessment of Surgical Skill Using Artificial Intelligence Hand Tracking in Cardiothoracic Training: A Feasibility Study

**DOI:** 10.1093/icvts/ivag048

**Published:** 2026-02-10

**Authors:** Morsal Atazadah, Mounir Bourass, Samuel A Max, Ivo M Cilon, J Wolter A Oosterhuis, Laurent N A Coopmans, Robert J M Klautz, Jerry Braun, Edris A F Mahtab

**Affiliations:** Department of Cardiothoracic Surgery, Leiden University Medical Center, Leiden, ZA 2333, The Netherlands; Department of Cardiothoracic Surgery, Leiden University Medical Center, Leiden, ZA 2333, The Netherlands; Department of Cardiothoracic Surgery, Leiden University Medical Center, Leiden, ZA 2333, The Netherlands; Department of Cardiothoracic Surgery, Leiden University Medical Center, Leiden, ZA 2333, The Netherlands; Department of Cardiothoracic Surgery, Leiden University Medical Center, Leiden, ZA 2333, The Netherlands; Department of Cardiothoracic Surgery, Leiden University Medical Center, Leiden, ZA 2333, The Netherlands; Department of Cardiothoracic Surgery, Leiden University Medical Center, Leiden, ZA 2333, The Netherlands; Department of Cardiothoracic Surgery, Amsterdam University Medical Center, Location Amsterdam Medical Center, Amsterdam, AZ 1105, The Netherlands; Department of Cardiothoracic Surgery, Leiden University Medical Center, Leiden, ZA 2333, The Netherlands; Department of Pulmonary Surgery, Amsterdam University Medical Center, Location Vrije Universiteit Medical Center, Amsterdam, HV 1081, The Netherlands; Department of Cardiothoracic Surgery, Leiden University Medical Center, Leiden, ZA 2333, The Netherlands

**Keywords:** surgical competency, artificial intelligence, hand tracking, technical skills, surgical training, skills assessment, cardiothoracic surgery

## Abstract

**Objectives:**

Measuring surgical competency is essential for surgical residents to ensure patient safety. Traditional assessment tools rely on subjective evaluation. This study evaluated whether artificial intelligence (AI)-based hand tracking can more objectively distinguish between levels of surgical competency and predict surgical years of experience versus traditional assessments.

**Methods:**

A total of 44 participants, including medical students, surgical residents, and surgical consultants, performed transcutaneous suturing, intracutaneous suturing, and surgical knot tying. Videos of intracutaneous suturing were scored using the objective structured assessment of technical skills (OSATS). Hand movements were analysed using AI tracking software to extract coordinates to measure velocity, pathlength, and jerk. Linear regression models predicted experience years using procedural time and OSATS in combination with hand tracking metrics.

**Results:**

Hand tracking metrics varied mainly between medical students and more experienced groups. Traditional assessment tools (procedural time, OSATS) could predict experience years during training, with an adjusted coefficient of determination (R^2^) ranging from 0.537 to 0.638, dependent on procedure type. Hand tracking variables identified multiple significant predictors for years of experience, with an adjusted R^2^ of 0.540-0.712, which outperformed the traditional tools in each procedure. Combining all assessment tools (time, OSATS, and hand tracking) gave the best predictive value, with an adjusted R^2^ ranging from 0.540 to 0.809, with velocity, pathlength, jerk, and acceleration as significant predictors.

**Conclusions:**

AI-based hand tracking provides a new method for objective, reproducible measures of surgical skills. Incorporating hand tracking metrics enhances prediction of surgical experience, and supports standardized as well as objective evaluation of skills assessment in surgical training.

## Graphical abstract

**Figure:**
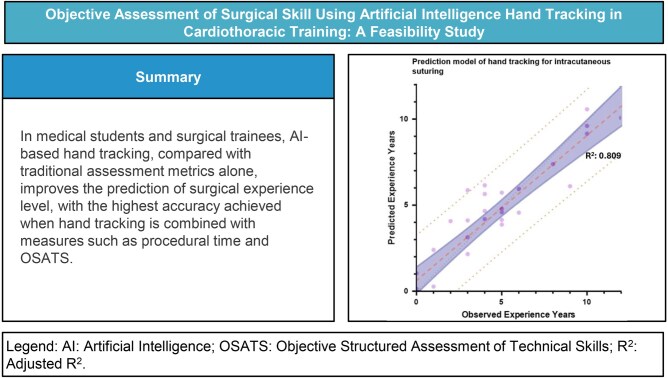

## INTRODUCTION

Developing proficiency in surgical technical skills is a core component of training, critical for both competency and patient safety. Traditionally, assessment has relied mainly on evaluations based on direct observation by supervising surgeons.^1^ One of the widely used assessment tools is the objective structured assessment of technical skills (OSATS), which evaluates performance using predefined domains such as tissue handling.^2^

While supervision and direct feedback from experienced surgeons remain essential in surgical training, the OSATS and similar methods rely on judgement of individual assessors, which may lead to inter-rater variability and potential bias.^3^ Furthermore, frequent assessment of technical performance can be time-consuming and usually requires the presence of supervisors both during and after procedures. These disadvantages show the need for more objective, scalable, and reproducible methods to assess surgical skills.

Objective parameters, such as complication rates, operative blood loss, and operative time, have been introduced to assess surgical proficiency.^4^ This is shown in the variety of variables used to evaluate learning curves and thereby, surgical proficiency.^4^^,^^5^ While these metrics are widely used and provide meaningful information, they are influenced by multiple factors, including patient complexity, comorbidities, and the surgeon’s level of experience. As a result, they may not consistently reflect technical skill alone.^6^^,^^7^ Similarly, case volume contributes to procedural exposure and has been associated with competency. However, case volume alone does not fully capture technical proficiency and should therefore be complemented by other performance-based assessments.^7^

Recent technological advancements have introduced the potential for objective approaches to skill assessment and training.^8^ Early research with instrument tracking provided only indirect information about a surgeon’s actions, and did not fully capture the nuances of fine hand movements. More recently, hand motion tracking applications have been explored.^8–10^ However, multiple studies limit their analysis to only a few hand tracking variables or to comparisons between the lowest and highest levels of experience, such as medical students and surgeons.^11^^,^^12^ As a result, little is known about how hand tracking metrics evolve across the intermediate stages of training, such as residents, or across each year of surgical training.

Artificial intelligence (AI)-based hand tracking offers the potential to evaluate surgical performance through multiple metrics.^11–14^ These metrics have been proposed as promising tools for objective skill evaluation. However, no study has developed a predictive model to estimate years of experience using hand tracking parameters. Such a model could reduce reliance on direct supervision, provide a standardized and objective way of skills assessment, and give insight into a trainees’ competencies and areas for improvement. One of the primary factors behind physician burnout is the administrative burden, which in some countries, including the Netherlands, accounts for approximately 40% of a physicians’ duties.^15^ AI tools may help reduce part of the workload associated with frequent assessments for both supervisors and trainees. Additionally, by supporting supervisors and providing trainees with clearer insight into their progress and areas for improvement, AI-based hand-tracking tools may help create a more efficient and supportive training environment. This could indirectly contribute to reducing burnout and drop-out among trainees and residents.

Cardiothoracic surgery involves technically demanding procedures with steep learning curves. Therefore, objective tools such as AI-based hand tracking may be especially valuable in this field by providing objective, consistent, reproducible metrics that support structured training, describing individuals learning curves and providing early identification of technical gaps.

The aim of this study is to determine whether AI-based hand tracking can objectively distinguish competence levels and to explore the feasibility of developing a predictive model for surgical experiences based on hand tracking, compared to traditional OSATS assessment and procedural time.

## METHODS

### Study design

This prospective study was conducted at Leiden University Medical Center (LUMC) and Amsterdam University Medical Center (AUMC). Approval was obtained from the institutional scientific board authorities (DAP/tak/0112025; 31-01-2025). Written informed consent was obtained from all participants prior to inclusion in the study. Participant data were stored and used in accordance with institutional policies and the WMA Declaration of Taipei.

### Participants

Participants (*n* = 44) were recruited from February 2025 until August 2025 and divided into 3 groups: medical students *n* = 11, residents *n* = 21, surgical consultants *n* = 12 (**Table 1**).

**Table 1.** Participant Characteristics

Experience level	N	Surgical department (% from cardiothoracic surgery)	Sex (% male)	Years of clinical experience (mean ± SD)
Medical student	11	N/A	57.1	1.3 ± 1.3
Surgical resident	21	66.7	76.1	6.7 ± 2.8
Surgical consultant	12	91.7	91.7	28.1 ± 7.7

### Procedure

Participants were recorded while performing 3 standard predefined surgical tasks on synthetic skin pads after a standardized explanation. The investigator was present throughout each task to ensure procedural consistency and answer questions. The tasks were selected to represent common basic suturing techniques in the following order:

- Interrupted suturing: 3 interrupted sutures placed on the incision, each tied using instruments.
- Predefined surgical knot manually tied after a single interrupted suture.
- Intracutaneous suturing: a continuous suture placed along an incision of 5.5 cm, finished with an Aberdeen knot.

Procedures were performed using standardized instruments: needle holder, anatomical forceps, suture scissors, Ethicon Prolene 2-0 SH and 4-0 RB-1 (Johnson and Johnson, Raritan, New Jersey, United States). Tasks were recorded from above the operating field using a Sony α7s mirrorless digital camera (Sony Group Corporation, Tokyo, Japan) with a Sony FE 28-70 mm F3.5-5.6 OSS lens, mounted to a metal frame. Tracking of the hands started when the needle entered the skin and ended when the excess suture wires were cut.

### Data collection and processing

Recordings were recorded at 1920 × 1080pixel resolution using the H.264 codec and processed with AI-based hand tracking software (MediaPipe 0.10.14). Coordinates were extracted for 21 anatomical landmarks per hand for every frame of the video. The tracking software recorded position data (x, y, z-coordinates) of each landmark per frame. From these coordinates, multiple motion metrics were calculated regarding surgical proficiency:

- Pathlength: The total distance moved by the hands during the procedure.
- Total jerk: A measure of the rate of change in acceleration of the hands movement over time.
- Dimensionless jerk: A proxy for smoothness of motion of the hands, calculated using the change in acceleration during the procedure.
- Average and peak velocity: The mean and maximum speed of hand movements.
- Average and peak acceleration: The mean and maximum rates of change in velocity.
- Procedural time: The duration from the first needle entry into the skin to the cutting of the excess suture material.

Kinematic analyses were performed in Python 3.11.9 using FastAPI (0.115.5), MediaPipe (0.10.14), OpenCV (4.10.0), NumPy (1.26.4), SciPy (1.13.1), and pandas (2.2.2). Hand landmarks (21 points per hand) were detected frame-by-frame using the MediaPipe Hand Landmarker (video mode, 2 hands). Trajectories were smoothed with a One-Euro filter (min_cutoff = 0.003, beta = 0.2; adjustable), which was adaptively scaled per landmark type (fingertips more strictly; joints moderately; palm/wrist more responsively).

Kinematics were computed on the 2 D palm centre (mean of landmarks 0, 5, 9, 13, 17), scaled to pixels, and differentiated according to the video framerate (dt = 1/FPS) into velocity, acceleration, and jerk. This subset was chosen as a stable proxy for overall hand motion, reducing variability from fingertip movements while maintaining representative kinematics. From these, cumulative pathlength, cumulative jerk, and peak and mean values were derived.

Outliers in the jerk signal were automatically detected and removed using an interquartile-range method (default threshold 1.30) or z-score; up to 10% of points could be removed, with automatic threshold relaxation if that percentage was exceeded. Missing values resulting from transient hand detection failures were handled using linear interpolation for gaps of up to 10 frames, while longer gaps were excluded; short gaps at the start or end of the sequence were addressed using forward- and backward-filling. Artifact segments could optionally be excluded after visual inspection, after which the analysis was rerun. Annotated output videos with landmarks were generated for quality control. Removed jerk outlier samples were excluded from subsequent kinematic derivative calculations and were not imputed.

### OSATS rating

A cardiothoracic surgeon, a thoracic surgeon, and a surgical resident independently scored the video fragments containing the intracutaneous closure. The 7-point Likert scale OSATS scoring was only performed for this specific procedure (**Table 2**).^2^

**Table 2.** Metrics of the Intracutaneous Suturing Task per Experience Level, Presented as Mean ± Standard Deviation

Column1		Medical students	Residents	Surgical consultant	*P*-value
Mean OSATS score		18.3 ± 6.0	36.0 ± 9.6	43.6 ± 2.9	<.001^*^
Procedural time		500.6 ± 136.8	233.7 ± 66.0	195.6 ± 47.0	<.001^*^
Total hand jerk	Non-dominant hand	4.90 × 10^5^ ± 2.00 × 10^5^	2.49 × 10^5^ ± 1.18 × 10^5^	2.17 × 10^5^ ± 1.33 × 10^5^	<.001^*^
	Dominant hand	4.46 × 10^5^ ± 1.47 × 10^5^	3.15 × 10^5^ ± 1.31 × 10^5^	2.41 × 10^5^ ± 1.30 × 10^5^	.007^*^
Log dimensionless hand jerk	Non-dominant hand	29.8 ± 2.5	26.0 ± 2.1	25.3 ± 1.3	<.001^*^
	Dominant hand	29.5 ± 1.5	27.3 ± 2.1	25.5 ± 3.2	.003^*^
Average hand acceleration	Non-dominant hand	288.7 ± 204.6	325.0 ± 160.0	881.0 ± 593.6	<.001^*^
	Dominant hand	573.1 ± 549.4	908.4 ± 1014.8	813.9 ± 625.1	.593
Peak hand acceleration	Non-dominant hand	1.17 × 10^5^ ± 8.29 × 10^4^	8.51 × 10^4^ ± 5.57 × 10^4^	1.72 × 10^5^ ± 9.49 × 10^4^	.022^*^
	Dominant hand	1.23 × 10^5^ ± 1.07 × 10^5^	1.24 × 10^5^ ± 1.01 × 10^5^	1.25 × 10^5^ ± 1.13 × 10^5^	.999
Pathlength	Non-dominant hand	2.41 × 10^4^ ± 1.04 × 10^4^	8.85 × 10³ ± 3.94 × 10³	8.73 × 10³ ± 5.59 × 10³	<.001^*^
	Dominant hand	2.00 × 10^4^ ± 8.36 × 10³	1.12 × 10^4^ ± 4.81 × 10³	9.26 × 10³ ± 4.03 × 10³	<.001^*^
Average velocity	Non-dominant hand	54.0 ± 24.0	51.1 ± 16.7	94.6 ± 42.6	<.001^*^
	Dominant hand	65.4 ± 36.7	89.0 ± 40.4	93.0 ± 41.7	.244
Peak velocity	Non-dominant hand	4.83 × 10³ ± 3.32 × 10³	3.33 × 10³ ± 2.33 × 10³	6.61 × 10³ ± 3.47 × 10³	.027^*^
	Dominant hand	4.92 × 10³ ± 4.28 × 10³	4.52 × 10³ ± 3.18 × 10³	4.22 × 10³ ± 3.47 × 10³	.912

*P*-values are based on MANOVA across the 3 groups.

* Indicates statistical significance (*P* < .05). Abbreviation: OSATS, objective structured assessment of technical skills.

### Statistical analysis

The distribution of the data was assessed using histograms and Q-Q plots. Variables that deviated from normality were natural log transformed prior to analysis. Descriptive statistics are reported as mean ± standard deviation (SD) for normally distributed variables.

To examine whether the 3 experience levels differ, a multivariate analysis of variance (MANOVA) was performed. MANOVA was used as an exploratory analysis to evaluate group-level differences across correlated performance metrics, while primary hypothesis testing focused on regression-based prediction of surgical experience. The variables used are OSATS, procedural time, velocity, acceleration, pathlength, dimensionless jerk, and total jerk. Tukey post-hoc analyses were performed to determine which groups differed significantly.

Linear regression analyses were performed separately for each procedure. For each procedure, multiple models were developed. The first model included procedural time (an extra model was developed for OSATS rating for intracutaneous suturing) as predictors of surgical experience. The second model incorporated hand tracking parameters. A backward selection was applied to identify the most relevant predictors. Model performance was evaluated using the adjusted coefficient of determination (R^2^). The R^2^ represents the proportion of variance in surgical experience explained by the model. Adjusted R^2^ corrects this value for the number of predictors to prevent overestimation when additional variables are included. Higher values indicate a better overall model fit.

### Predictive model

Initial visual inspection of the data demonstrated a plateau in hand tracking metrics at the level of surgical consultants, suggesting limited additional improvement in basic surgical skills with increasing experience. As the objective of this study was to evaluate whether hand tracking metrics could predict years of training, only participants currently in training were included in the regression analysis. Consequently, surgical consultants were excluded. Experience years were recorded as the total duration of clinical training. To account for variation in competency within medical students, clinical rotations were included in this calculation: early-year rotations were weighted as equivalent to 1 year of experience, whereas final-year rotations were weighted as 3 years, reflecting their closer proximity to physician-level proficiency.

## RESULTS

### OSATS scores and procedural time

Across the experience groups, mean OSATS were given to the intracutaneous suturing fragments. MANOVA revealed a significant overall effect of experience level on both OSATS ratings and procedural duration (*P* < .001, **Table 2**). **Table 2** presents the descriptive statistics (mean ± SD) for each participant group alongside *P*-values derived from the MANOVA model. Means and standard deviations were calculated directly per group, while all *P*-values were obtained from the MANOVA. Post-hoc Tukey analyses showed that the OSATS scores significantly increased with each experience group. Procedure time significantly decreases with each experience group, with no significant differences between residents and surgical consultants.

### Difference of hand tracking between groups

A 1-way MANOVA was conducted to examine the effect of hand tracking metrics on experience level. The multivariate effect during the knot tying (Pillai’s Trace: 0.959, F: 1.38, p: 0.168), and interrupted sutures (Pillai’s Trace: 1.007, F: 1.62, p: 0.066) were not significant. The multivariate effect of experience during the intracutaneous suturing task (Pillai’s Trace: 1.406, F: 3.10, *P* <.001) was significant. Post-hoc Tukey HSD tests showed that medical students generally had higher total jerk and higher pathlength compared to more experienced participants, whereas surgical consultants exhibited higher velocity- and acceleration-related measures than residents.

### Predictive model

**Figure 1.**
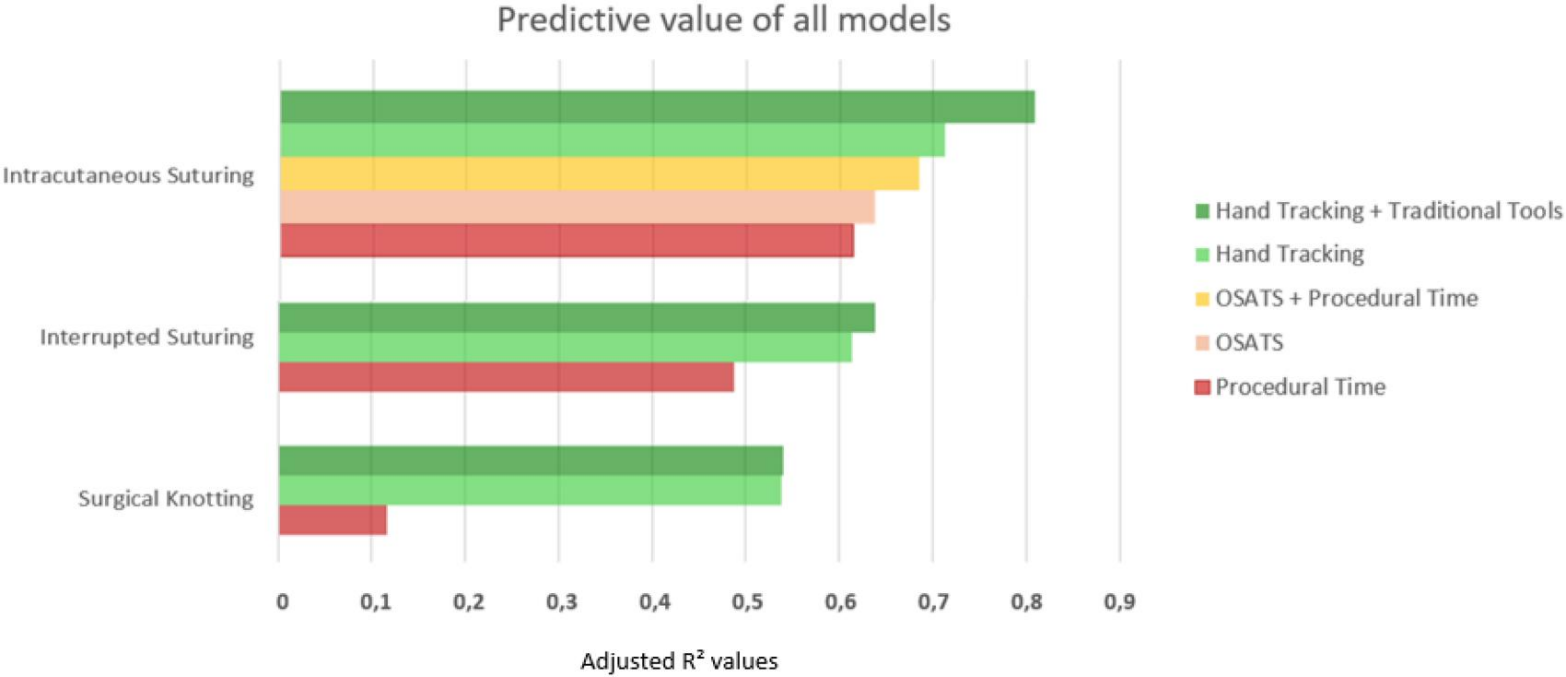
Adjusted R^2^ Values of Predictive Models for Surgical Performance Across 3 Tasks. Models included objective structured assessment of technical skills (OSATS scores), procedural time, hand tracking metrics, and hand tracking combined with traditional tools. The x-axis shows the adjusted R^2^ values, which indicates the proportion of variance in observed experience explained by the model. Higher values indicate greater explained variance in performance. Abbreviation: OSATS, objective structured assessment of technical skills

**Figure 2.**
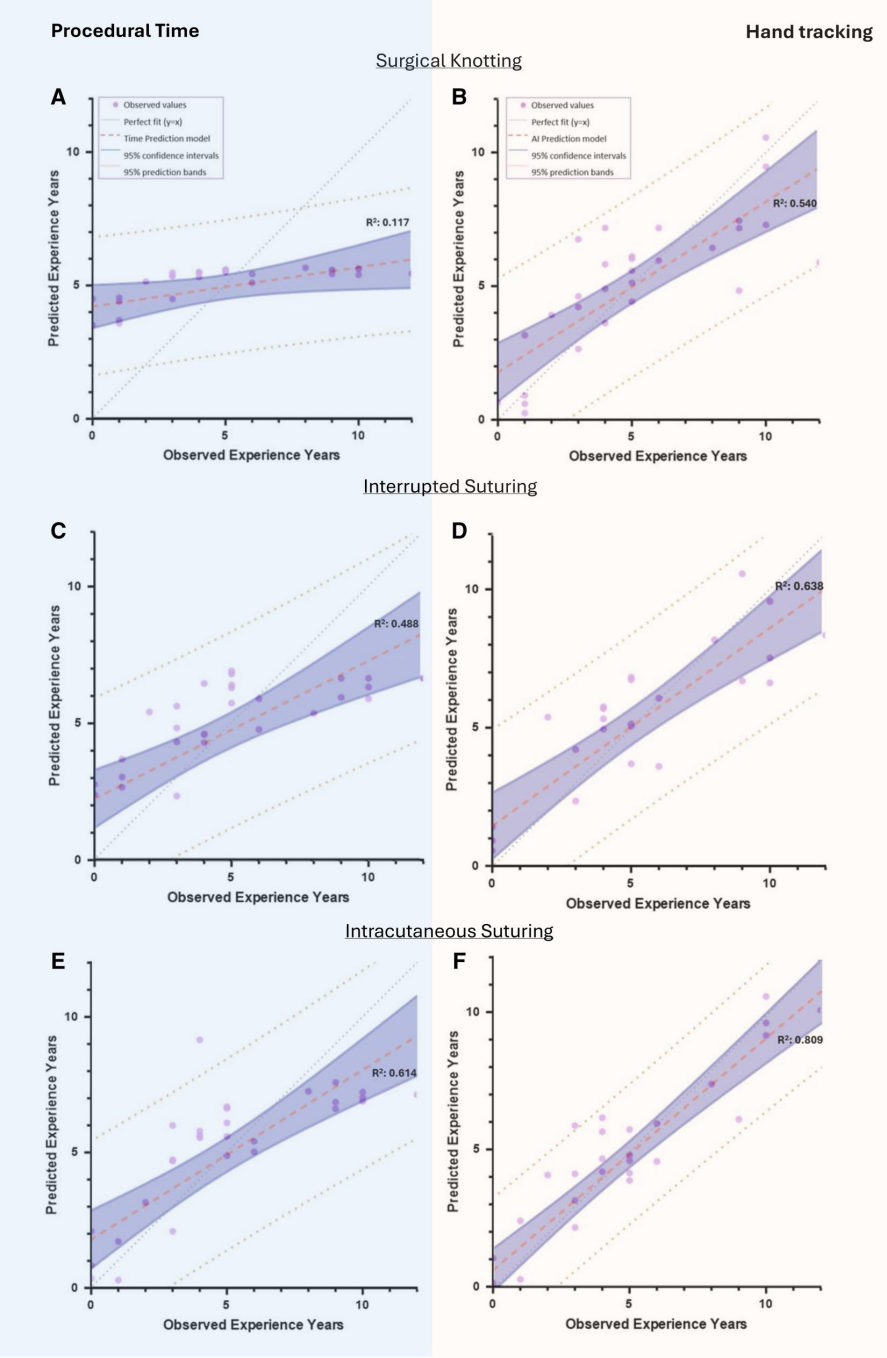
Observed-versus-Predicted Surgical Experience Plots Across Different Tasks. Graphs A, C, and E show predictive models based on procedural time for surgical knotting (A), interrupted suturing (C), and intracutaneous suturing (E). Graphs B, D, and F show predictive models incorporating hand tracking data with traditional assessment tools for surgical knotting (B), interrupted suturing (D), and intracutaneous suturing (F). AI prediction model refers to artificial intelligence prediction model. The x-axis represents observed experience years, the y-axis represents predicted experience years. The coefficient of determination (R^2^) indicates the proportion of variance in observed experience explained by the model. Across all tasks, inclusion of hand tracking data improves predictive accuracy. Abbreviation: AI, artificial intelligence

For surgical knot tying, the baseline model containing procedural time had an adjusted R^2^ of 0.117. The predictive model with hand tracking parameters had an adjusted R^2^ of 0.537, meaning that almost 54% of the variance in experience could be explained by hand tracking (Figure 1). Combining both assessment tools resulted in an adjusted R^2^ of 0.540, with pathlength, acceleration and procedural time as significant predictors. These results indicate that hand tracking metrics capture substantial variance in skill level beyond procedural time alone (**Figure 2A and B**).

For the interrupted suturing task, the baseline model with procedural time had an adjusted R^2^ of 0.488. The model including only hand tracking variables had an adjusted R^2^ of 0.614. Combining hand tracking parameters with procedural time increased the adjusted R^2^ to 0.638, with procedural time, velocity and acceleration as significant predictors. These results indicate that hand tracking metrics provide additional objective information beyond conventional assessment tools such as procedural time (**Figure 2C and D**).

In intracutaneous suturing, procedural time had an adjusted R^2^ of 0.614, while mean OSATS scores had an adjusted R^2^ of 0.638. Hand tracking variables identified multiple significant predictors for experience years, with an adjusted R^2^ of 0.712. Combining all 3 assessment tools produced a R^2^ of 0.809, with OSATS, jerk, pathlength, velocity, and acceleration remaining significant. These results indicate that, especially in more time-consuming tasks, hand tracking metrics capture subtle differences in skill level that are not fully reflected in OSATS or procedural time (**Figures 1 and 2E and F**).

## DISCUSSION

This study investigated the feasibility of using AI-based hand tracking to objectively assess surgical skills across different experience levels and to develop a predictive model using hand tracking parameters to predict experience. This is the first predictive model for estimating surgical experience with hand tracking metrics.^1^^,^^3^

### Differences across experience levels

Significant differences were observed during intracutaneous suturing in procedural time, OSATS, jerk and pathlength between medical students and other levels, with higher expertise associated with faster, more efficient, and smoother movements. Furthermore, differences were also seen between surgical consultants and other experience levels when performing intracutaneous suturing in acceleration and velocity. No significant differences were found for surgical knot tying or interrupted suturing. These findings are supported by Genovese et al,^11^ showing no differences in motion tracking between cardiothoracic surgeons and residents who completed 1-2 clinical years.

Both surgical knot tying and interrupted suturing are relatively short tasks, resulting in relatively short video segments that provide limited movement data for analysis, which are relatively simple, highly standardized, and practiced early in training. This may reduce the ability to detect differences between groups. Furthermore, surgical knot tying and subtle technical elements of suturing, such as needle entry angle and rotational passage through tissue, rely on fine motor control of the fingers and wrist. Because our kinematic analysis was based on palm trajectories, this method is more sensitive to global rather than fine motor motion. Therefore, the applied hand-tracking metrics may not fully capture all aspects of technical performance, which could explain the absence of significant group differences observed.

Another consideration is that surgical proficiency may not fit precisely into categorical groups such as resident and surgeon. As shown in **Table 2**, significant differences were best seen between medical students and more experienced participants, whereas differences between residents and surgical consultants were less consistent. Previous studies have largely focused on extremes, such as medical students and fully trained surgeons.^12^^,^^13^ By contrast, our predictive model shows that analysing hand tracking metrics by training years may provide a more sensitive, individualized measure of surgical proficiency than fixed groups, suggesting that training years, rather than categories, are better captured through objective motion-based metrics.

### Predictive model

Regression analyses show that predictive models incorporating hand tracking variables explained surgical experience more accurately than conventional assessment tools, like the OSATS and procedural time alone. A recent systematic review in cardiothoracic surgery reported that the OSATS was the most frequently applied assessment tool, used in 61% of the studies, and procedural time in 60%.^4^ Another systematic review of learning curve studies in cardiothoracic and vascular surgery found that operative time was reported in >95% of studies, although it was understood to have limited correlation with patient outcomes.^6^ This shows that current assessment of skills relies mainly on the OSATS and timing. However, the predictive value of this traditional model was lower than the models incorporating hand tracking metrics, highlighting the added benefit of AI-based objective measures. In all 3 procedures, the addition of hand tracking variables resulted to a better predictive model. This demonstrates that AI-based hand tracking adds measurable value to existing assessment methods, rather than replacing them. Hand tracking provides objective, reproducible measures of performance, which may enhance the monitoring of learning curves and support the delivery of targeted feedback to trainees. This technology could be integrated into national assessment platforms and digital portfolios to objectively assess surgical dexterity against reference standards or peer performance, and to monitor progression over time. This may support the selection process of new trainees and surgical training by providing objective, reproducible feedback, with the potential of longitudinal monitoring of technical development to describe learning curves. Such objective metrics can support supervisors by providing structured insights, but do not replace expert judgement by supervisors.

Future research should validate these findings in larger cohorts, assess variability within experience groups and investigate the applicability of predictive models for describing learning curves throughout residency training. Additionally, the utility of hand tracking in more complex surgical procedures or in operative settings should be explored as well.

### Limitations

Procedures were performed on synthetic skin pads in a simulated setting. While ensuring consistency, this does not fully capture the complexity, variability, and stressors of real surgical cases. Moreover, AI-based hand tracking evaluates selected objective aspects of technical performance, such as movement efficiency and smoothness. Other qualitative parameters including tissue handling, ergonomics, and assistance are not measured. Additionally, surgical consultants were excluded from regression analyses, as the data demonstrated a plateau and we aimed to analyse the predictive value of hand tracking on training years. This likely reflects the ceiling effect of basic suturing skills, which may not be sufficiently challenging to differentiate between residents and surgical consultants.^16^ Furthermore, in several cases the application initially failed to track hands. To include these data, the minimal detection confidence was empirically lowered to 0.55. While this theoretically increases the chance of false positives, all data were visually inspected, and no misclassifications were observed.

## CONCLUSION

AI-based hand tracking provides an objective method to assess the studied surgical technical skills and distinguish between different experience levels. In our study, AI-based hand tracking outperforms the traditional assessment methods in predicting surgical experience level. When combined with traditional metrics such as OSATS and procedural time, hand tracking metrics improve the prediction of surgical proficiency, particularly in trainees from medical student to resident level.

## Data Availability

The data underlying this article will be shared on reasonable request to the corresponding author.

## References

[1] Van Hove PD , Tuijthof GJM, Verdaasdonk EGG, Stassen LPS, Dankelman J. Objective assessment of technical surgical skills. Br J Surg. 2010;97:972-987. 10.1002/bjs.7115 20632260

[2] Martin JA , Regehr G, Reznick R, et al Objective structured assessment of technical skill (OSATS) for surgical residents. Br J Surg. 1997;84:273-278. 10.1046/j.1365-2168.1997.02502.x 9052454

[3] Hatala R , Cook DA, Brydges R, et al Constructing a validity argument for the objective structured assessment of technical skills (OSATS): a systematic review of validity evidence. Adv Health Sci Educ Theory Pract. 2015;20:1149-1175. 10.1007/s10459-015-9593-1 25702196

[4] Hussein N , Van Den Eynde J, Callahan C, et al The use of objective assessments in the evaluation of technical skills in cardiothoracic surgery: a systematic review. Interact CardioVasc Thorac Surg. 2022;35. 10.1093/icvts/ivac194 PMC9403301 35900153

[5] Chahal B , Aydin A, Amin MSA, et al The learning curves of major laparoscopic and robotic procedures in urology: a systematic review. Int J Surg. 2023;109:2037-2057. 10.1097/js9.0000000000000345 37132184 PMC10389344

[6] Arora KS , Khan N, Abboudi H, et al Learning curves for cardiothoracic and vascular surgical procedures—a systematic review. Postgrad Med. 2015;127:202-214. 10.1080/00325481.2014.996113 25529043

[7] Stride HP , George BC, Williams RG, et al Relationship of procedural numbers with meaningful procedural autonomy in general surgery residents. Surgery. 2018;163:488-494. 10.1016/j.surg.2017.10.011 29277387

[8] Yangi K , On TJ, Xu Y, et al Artificial intelligence integration in surgery through hand and instrument tracking: a systematic literature review. Front Surg. 2025;12:1528362. 10.3389/fsurg.2025.1528362 40078701 PMC11897506

[9] Arnold S , Svendsen M, Konge L, et al Three-dimensional motion tracking correlates with skill level in upper gastrointestinal endoscopy. Endoscopy. 2015;47:825-828. 10.1055/s-0034-1391884 25826273

[10] Lyon SM , Zeng W, Yang S, et al Microsurgery in motion: an objective assessment of microsurgical skill and efficiency. J Reconstr Microsurg. 2025;41:684-692. 10.1055/a-2491-3249 39814035

[11] Genovese B , Yin S, Sareh S, et al Surgical hand tracking in open surgery using a versatile motion sensing system: are we there yet? Am Surg. 2016;82:872-875. 10.1177/000313481608201002 27779963

[12] Hillemans V , Van De Mortel X, Buyne O, et al Objective assessment for open surgical suturing training by finger tracking can discriminate novices from experts. Med Educ Online. 2023;28:2198818. 10.1080/10872981.2023.2198818 37013910 PMC10075519

[13] Kholinne E , Gandhi MJ, Adikrishna A, et al The dimensionless squared jerk: an objective parameter that improves assessment of hand motion analysis during simulated shoulder arthroscopy. BioMed Res Int. 2018;2018:7816160-7816168. 10.1155/2018/7816160 30105247 PMC6076914

[14] Ebina K , Abe T, Hotta K, et al Automatic assessment of laparoscopic surgical skill competence based on motion metrics. PLoS One. 2022;17:e0277105. 10.1371/journal.pone.0277105 36322585 PMC9629630

[15] Kallewaard M , Van Schoten S, Trompert A, et al *Administratiedruk Medisch Specialisten*. 2017. Accessed December 12, 2025.https://demedischspecialist.nl/sites/default/files/20171117_DEF%20Rapport-administratiedruk-specialisten.pdf

[16] Munz Y , Moorthy K, Bann S, Shah J, Ivanova S, Darzi A. Ceiling effect in technical skills of surgical residents. Am J Surg. 2004;188:294-300. 10.1016/j.amjsurg.2004.02.006 15450837

